# Ovarian metastases from gallbladder mimics primary ovarian neoplasm in young patient: a case report

**DOI:** 10.1186/s13104-018-3283-z

**Published:** 2018-03-20

**Authors:** Ting-Ying Lee, Chia-Wen Wang, Teng-Wei Chen, De-Chuan Chan, Guo-Shiou Liao, Hsiu-Lung Fan

**Affiliations:** 1 Division of General Surgery, Department of Surgery, Tri-Service General Hospital, National Defense Medical Center, 325 Cheng-gong Rd Sec 2, Nei-hu, 114, Taipei, Taiwan, ROC; 2 Division of Plastic Surgery, Department of Surgery, Tri-Service General Hospital, National Defense Medical Center, 325 Cheng-gong Rd Sec 2, Nei-hu, 114, Taipei, Taiwan, ROC

**Keywords:** Gallbladder cancer, Ovarian invasion, Krukenberg tumor, Case report

## Abstract

**Background:**

Gallbladder cancer is unusually seen but can result in highly mortality rate. It makes challenge to diagnose for clinicians due to present asymptomatic or non-specific clinical presentation including abdominal pain, anorexia. It usually also accompanies with cholelithiasis (incidence is 1–2%) and incidentally detected by radiologic examination such as ultrasound, computed tomography or intra-operative intervention accidentally. Gallbladder cancer results in highly fatal malignancy because it is difficult to early detect. The ovarian metastases from gallbladder mimics primary neoplasm isn’t seen before and mentioned in English literatures before.

**Case presentation:**

A 28-year-old woman suffered from intermittently lower abdominal tenderness and nausea after meals for 3 years. The abdominal ultrasound revealed a right ovarian mass with fluid accumulation and the contrast CT of abdomen revealed a gallbladder fundus mass and liver tumor lesion located at segment 4. We arranged surgical intervention with radical cholecystectomy and debulking operation with salpingo-oophorectomy. The pathologic report revealed adenocarcinoma of gallbladder with liver, peritoneum, and right ovarian invasion. After surgical intervention, she also received adjuvant chemotherapy with Gemcitabine, Cetuximab, Cisplatin and Cyberknife.

**Conclusion:**

The non-specific symptoms make the challenge to difference the primary malignant neoplasm. The rarely diagnosis must take in consider if the gastrointestinal tract tumours coexist with ovarian tumours.

## Background

Carcinoma of the gallbladder is an incidental finding in 1% of patients undergoing cholecystectomy because of presenting cholelithiasis [1]. Most gallbladder cancers are adenocarcinoma (incidence was 70–90%) [2]. Patients usually present biliary symptoms or incidentally detect in abdominal viscera examination. The long-term outcomes are poor because the gallbladder cancer is an advanced disease when it presents symptoms.

The ovary is a site of metastasis for gastrointestinal neoplasms. It is known that gastric origin metastases to ovary as Krukenberg tumor [3]. The adenocarcinoma of gallbladder with ovary spread is quite rarely description in English literatures [2, 4, 5]. Here we present a 28-year-old woman presented lower abdominal pain. A right ovarian tumor and a gallbladder tumor were incidentally found in abdominal radiologic examinations. We arranged radical cholecystectomy and debulking operation with salpingo-oophorectomy bilateral, biopsy of the left ovary, and supra-colic omentectomy. The pathologic report proved that it was a neoplasm with multiple metastasis and the origin came from gallbladder. After receiving the surgical intervention, she underwent chemotherapy and Cyberknife for neoadjuvant therapy. The followed outcome was well and no immediately complication was noted.

## Case presentation

**Fig. 1:**
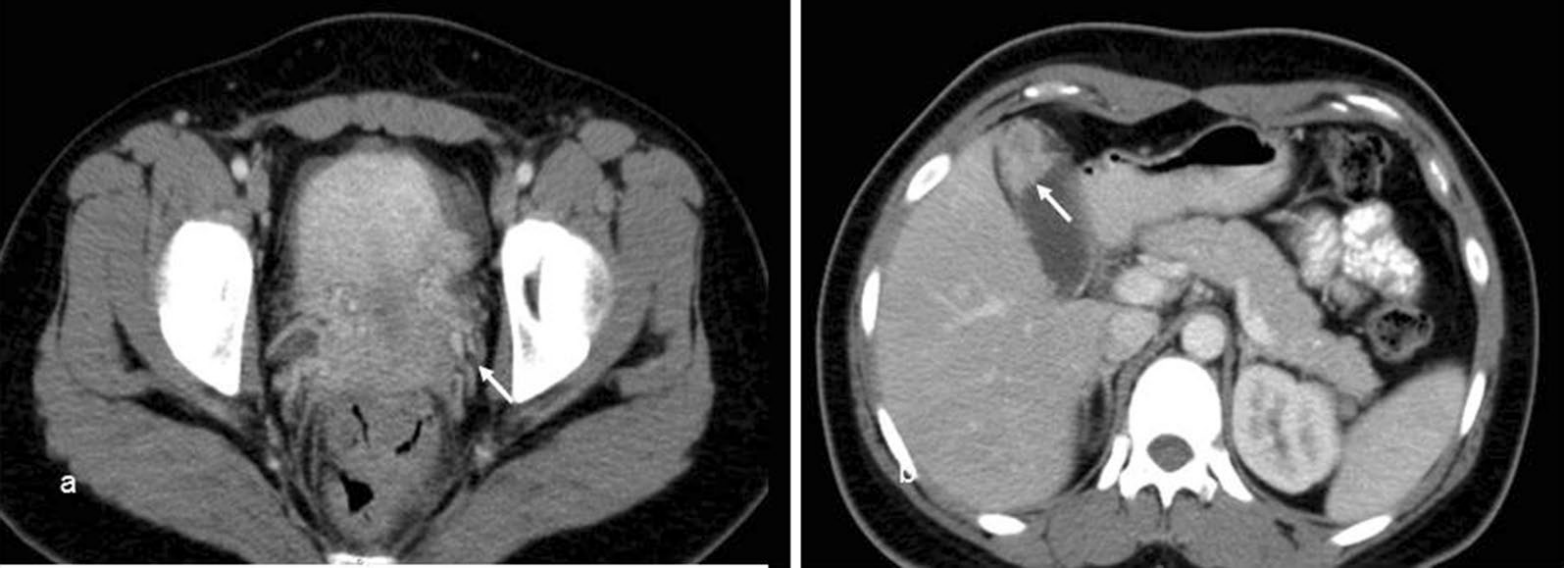
Contrast abdominal CT. **a** An irregular margin tumor located at right ovary (arrow). **b** A irregular tumor lesion enhanced and located at gallbladder fundus (arrow). But no symptom of extra-capsule invasion

**Fig. 2:**
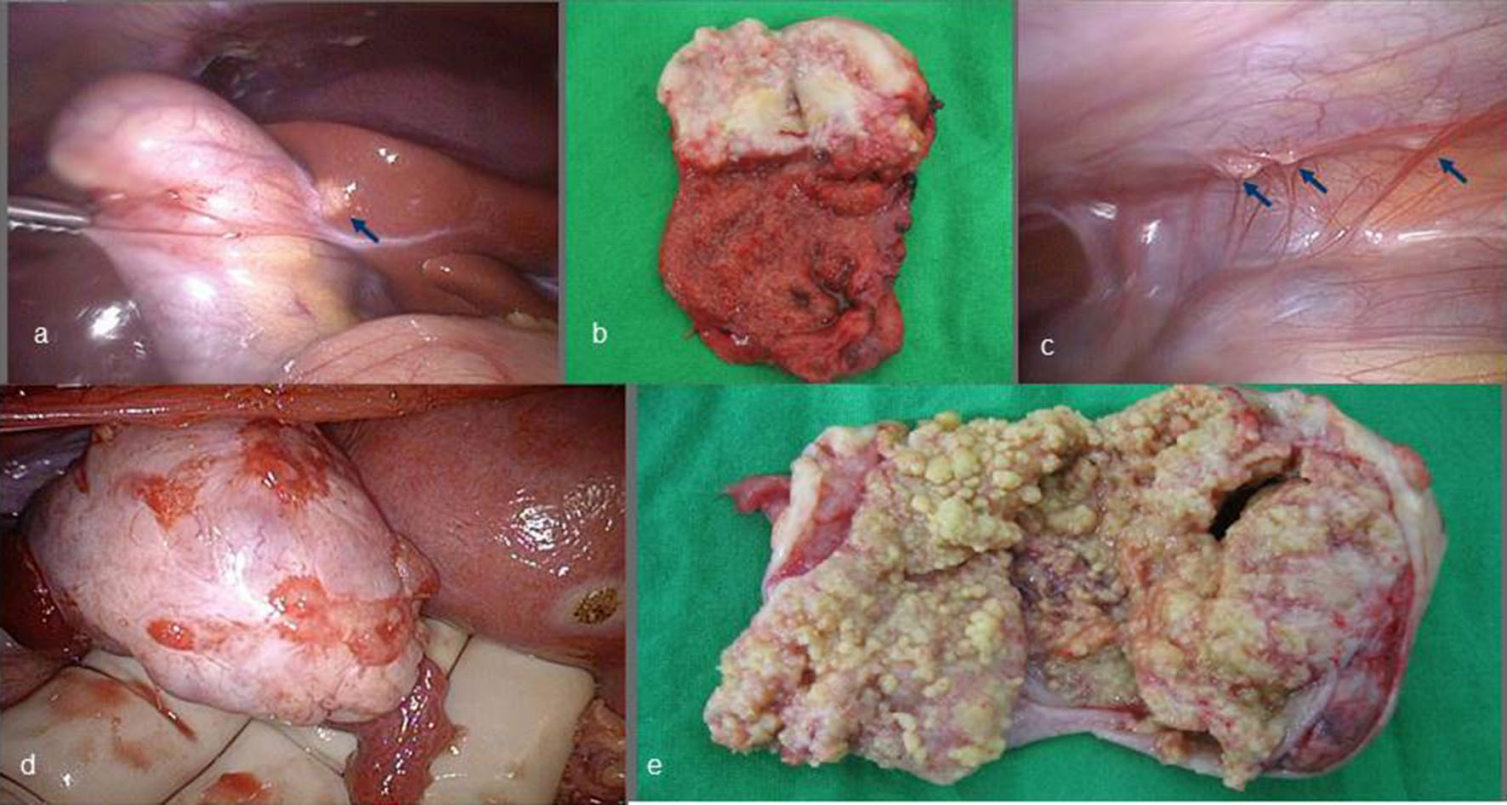
**a** Regular bladder shape with a small lesion over liver bed behind the gallbladder (arrow). **b** A polypoid tumor lesion located at gallbladder. **c** Several ill-defined lesions over abdominal wall was also found, carcinomatosis was suspected (arrows). **d** Right ovary was enlarged with irregular shape and **e** adenomyosis-like lesion occupied in the ovary

The 28-year-old woman complained intermittently lower abdominal pain combined with cold sweating and relieved after vomiting for 3 years. The painful sensation was description that lower abdominal tenderness without rebounding pain. There was no difficulty in urination. She had single sexual partner and no history of pregnancy. She didn’t take any oral contraceptives and regular menstruation. There was no dysmenorrhea and menorrhagia found. Her physical examination didn’t find obvious abdominal mass lesion and previous surgical scar. The labor test revealed normal result expect for mild elevated aspartate aminotransferase and alanine aminotransferase (AST/ALT: 128/132 U/L). Her abdominal ultrasound revealed a well-capsulized cystic lesion about 6 cm in diameter located at right ovary and mild fluid accumulated at pelvic region. The contrast abdominal CT also revealed a tumor lesions (2.3 × 3 × 2.1 cm in size) located at fundus of gallbladder with adjacent wall thickening and one small contrast decreased lesion located at segment 4 of liver (Fig. 1a–d). Initially ovary cancer with gallbladder and liver metastasis was impressed. But serum tumor marker was in normal range (AFP: 2.27 U/mL, CA-125: 27.51 U/mL, CEA: < 0.3 U/mL). She underwent laparoscopic cholecystectomy and wedge resection of liver nodule, segment 4 and debulking operation with salpingo-oophorectomy bilateral, biopsy of the left ovary, and supra-colic omentectomy. During the operative period, smooth shape gallbladder and right ovary were seen and resected (Fig. 2a–e). There was no obvious irregular surface performed on the resected gallbladder and ovary. There were 2 nodules located at upper abdominal wall. We did nodules resection and sent to frozen pathologic examination. The pathologic report confirmed it was abdominal carcinomatosis. The gallbladder and right ovary were explored and there were fixed, dense, adenomatosis hypertrophy seen inside. The finally pathologic report revealed that the gallbladder was the original source by two pathologists. The accurate diagnosis was adenocarcinoma of gallbladder with liver, abdominal wall and right ovary invasion. Now she starts to undergo following chemotherapy with Paclitaxel and Cisplatin. Unfortunately, after followed up for 2 years, she was expired due to carcinomatosis.

## Discussion and conclusion

This case that we misinterpreted metastatic spread to ovary as primary ovarian neoplasm is likely to occur when the ovarian lesion was the first manifestations of disease. Because of metastasis to the ovary from gallbladder carcinoma is rare. It spread by the way of abdominal cavity seeding and metastasis to ovary because of the relatively increased vascularity. This secondary ovarian carcinoma spread from gastrointestinal tract tumour is known as Krukenberg tumor.

Accurate distinction of the primary lesion from secondary ovarian tumour on the basis of imaging is difficult. According to Brown et al. [6] most metastatic ovarian neoplasms are predominantly solid or mixture of solid and cystic area. They also tend to be bilateral secondary ovarian neoplasm more often than unilateral primary neoplasm. Kim et al. [7] also suggested that secondary ovarian neoplasm should take in consider that solid ovarian tumours contain well demarcated intraluminal cystic lesions. In our patient, contrast CT revealed solid tumour within well ovoid surface gallbladder wall and the completely serosa layer was also confirmed during the surgical intervention.

**Fig. 3:**
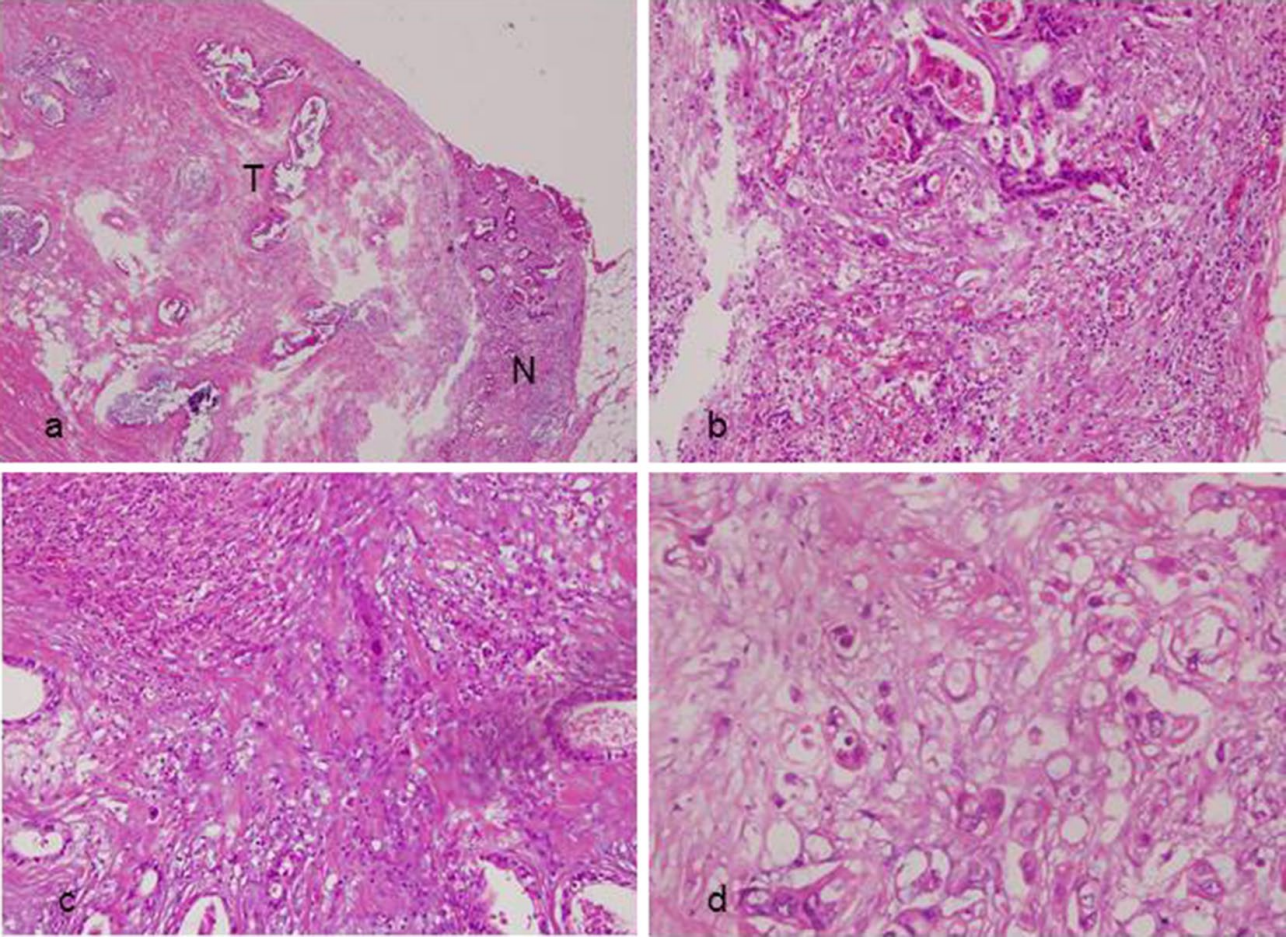
**a** Gallbladder tumor (T) under H&E stain revealed extra-capsule invasion of the gallbladder, and the margins is well identified to the normal cortex (N). **b** Irregular mucous gland infiltration with nuclear atypia in gallbladder lesion. There were multiple signet ring cells found. **c** Right ovary: mucous gland hyperplasia with cells atypia. **d** Right ovary: signet ring cells can be identified

**Fig. 4:**
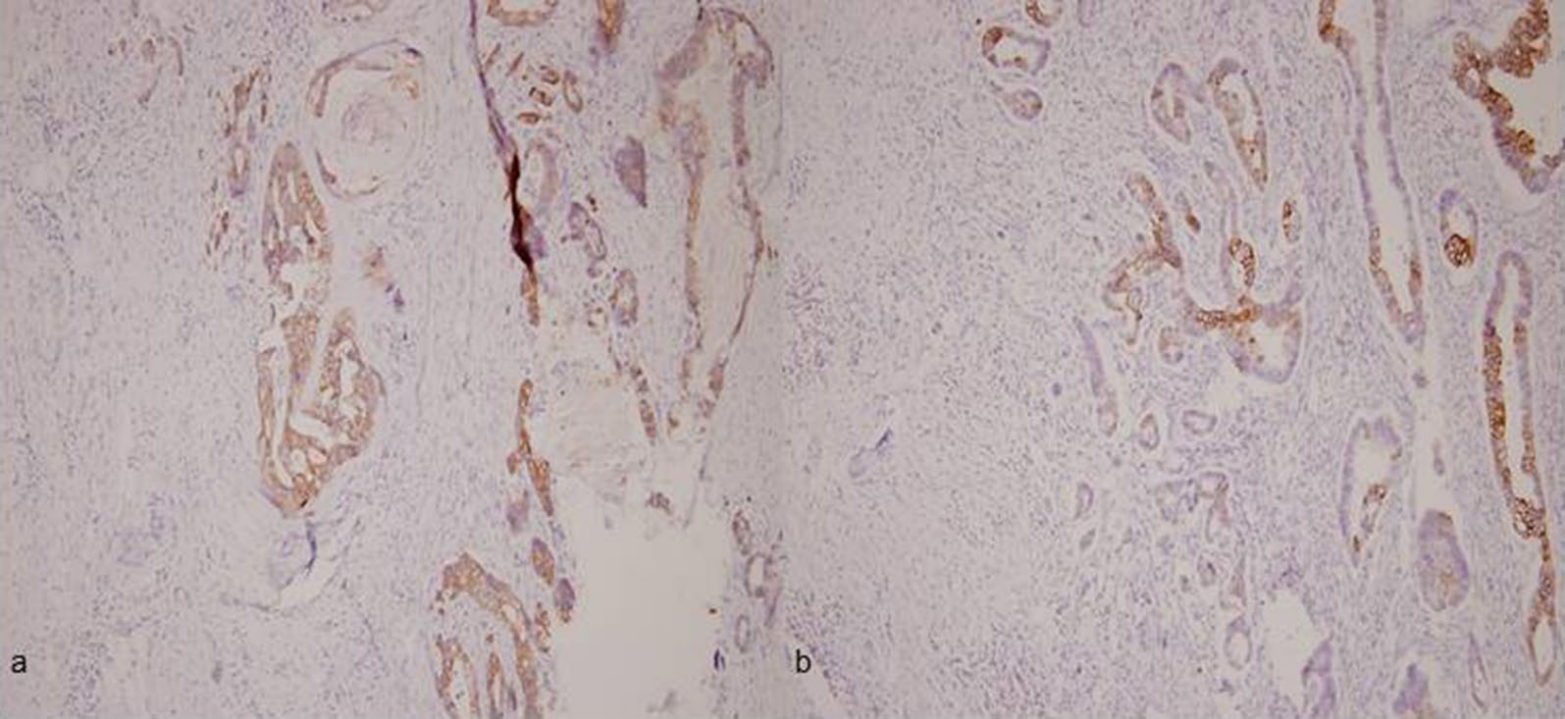
**a** Gallbladder tumor under CK-7 stain performed gland-forming adenocarcinoma. **b** Gallbladder cancer under CK-20 stain performed mucus hyperplasia

Operative frozen analysis also makes difficult to distinguish the primary neoplasm because the metastatic mucinous tumors can be very similar to primary ovarian mucinous tumors grossly and occasionally pose major diagnostic problems in microscopic appearance [4]. Utilized hematoxylin and eosin-stain (Fig. 3a–d) and immunohistochemical stain including CK-7 and CK-20 stain (Fig. 4a–b) can well identify goblet cells and signet-ring cells in primary gastrointestinal tract cancers [8, 9]. In concluded, the definitely method to evaluate the primary lesions metastasized to ovary depend on permanent pathology.

The management of this malignant neoplasm includes cytoreduction surgery (CRS). There were several literatures presented that hyperthermic intraperitoneal chemotherapy (HIPEC) post CRS may improve survival [10]. This patient may decrease recurrence if treated with CRS plus HIPEC. In conclusion, this is the youngest patient with gallbladder adenocarcinoma with ovarian invasion in the worldwide literatures that we had reviewed [4]. The presented non-specific symptoms make our diagnosis challenge and there is no effective method to approach the primary lesion. However, the rarely diagnosis must take in consider if the gastrointestinal tract tumours coexist with ovarian tumours.

## Abbreviations

CT: computed tomography
AST: aspartate aminotransferase
ALT: alanine aminotransferase
AFP: alpha-fetoprotein
CA-125: carbonhydrate antigen 125
CEA: carcinoembryonic antigen
CK-7: cytokeratin-7
CK-20: cytokeratin-20
H&E stain: hematoxylin and eosin stain
